# Expansion of human amniotic epithelial cells using condition cell reprogramming technology

**DOI:** 10.1007/s13577-022-00849-4

**Published:** 2022-12-31

**Authors:** Aisha Naeem, Muhammad Umer Choudhry, Alex Kroemer, Simone Wahnschafft, Wanxing Cui, Chris Albanese

**Affiliations:** 1 Department of Oncology, Lombardi Comprehensive Cancer Center, Georgetown University Medical Center, Washington, DC, USA; 2 Health Research Governance Department, Ministry of Public Health, Qatar, Saudi Arabia; 3 Center for Translational Transplant Medicine, MedStar Georgetown Transplant Institute, MedStar Georgetown University Hospital, Georgetown University, Washington, DC, 20007 USA; 4 MedStar Georgetown University Hospital, Washington, DC, USA; 5 Department of Radiology, Georgetown University Medical Center, Washington, DC USA; 6 Center for Translational Imaging, Georgetown University Medical Center, Washington, DC, USA

**Keywords:** Amniotic stem cells, Amniotic epithelial stem cells, Cell culture, Human serum, Stem cell markers

## Abstract

Human amniotic epithelial cells (hAECs) are non-immunogenic epithelial cells that can develop into cells of all three germline lineages. However, a refined clinically reliable method is required to optimize the preparation and banking procedures of hAECs for their successful translation into clinical studies. With the goal of establishing standardized clinically applicable hAECs cultured cells, we described the use of a powerful epithelial cell culture technique, termed Conditionally Reprogrammed Cells (CRC) for ex vivo expansion of hAECs. The well-established CRC culture method uses a Rho kinase inhibitor (Y-27632) and J2 mouse fibroblast feeder cells to drive the indefinite proliferation of all known epithelial cell types. In this study, we used an optimized CRC protocol to successfully culture hAECs in a CRC medium supplemented with xenogen-free human serum. We established that hAECs thrive under the CRC conditions for over 5 passages while still expressing pluripotent stem markers (OCT-4, SOX-2 and NANOG) and non-immunogenic markers (CD80, CD86 and HLA-G) suggesting that even late-passage hAECs retain their privileged phenotype. The hAECs-CRC cells were infected with a puromycin-selectable lentivirus expressing *luciferase* and *GFP (green fluorescent protein)* and stably selected with puromycin. The hAECs expressing GFP were injected subcutaneously into the flanks of Athymic and C57BL6 mice to check the tolerability and stability of cells against the immune system. Chemiluminescence imaging confirmed the presence and viability of cells at days 2, 5, and 42 without acute inflammation or any tumor formation. Collectively, these data indicate that the CRC approach offers a novel solution to expanding hAECs in humanized conditions for future clinical uses, while retaining their primary phenotype.

**Graphical abstract:**

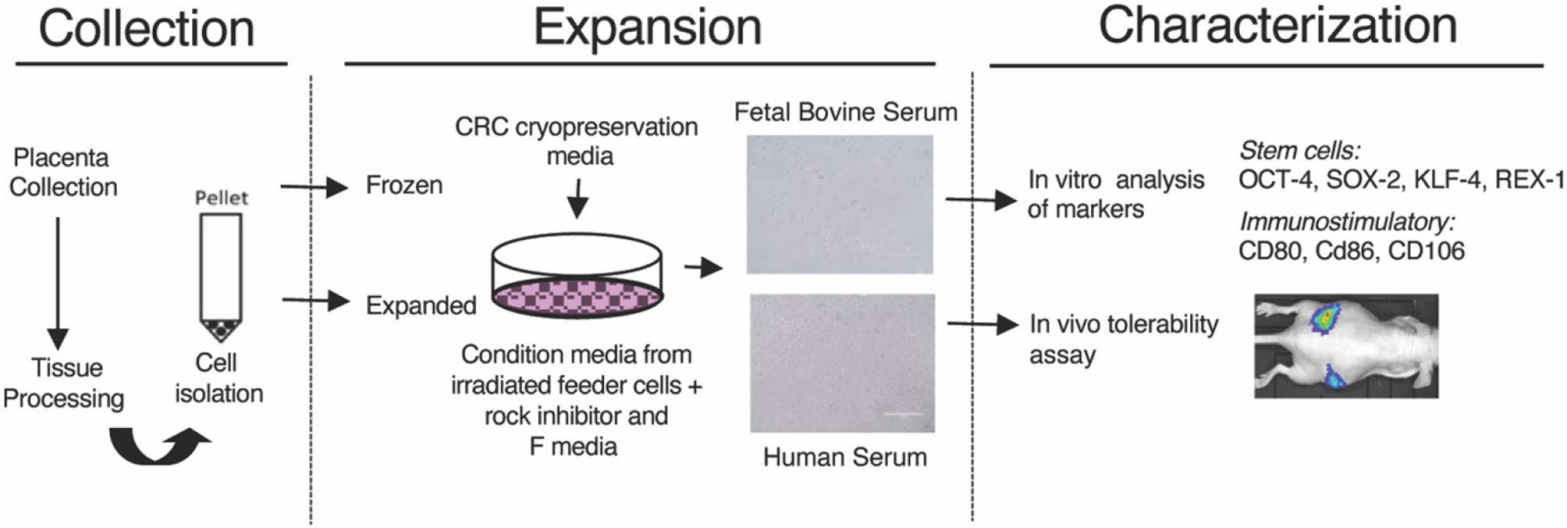

**Supplementary Information:**

The online version contains supplementary material available at 10.1007/s13577-022-00849-4.

## Introduction

Human amniotic epithelial cells (hAECs), originating from pluripotent placental epiblasts, maintain multilineage differentiation potential [1, 2]. hAECs are immune privileged and are capable of modulating the local immune response and demonstrate the exciting potential for therapeutic application in tissue regeneration [3, 4]. Despite the remarkable stem-like pluripotency of hAECs and ease of collection, as well as ethical considerations, the use of these cells is still restricted due to in part inefficient and limited ex vivo expansion [5]. To date, many protocols have been published to culture hAECs; however, an inability to scale and expand hAECs without significantly compromising their unique and privileged nature precludes the widespread use of hAECs for regenerative therapies [6]. This limitation is primarily due to the use of sub-optimal culture conditions that limit expansion through the induction of immunostimulatory markers, a concomitant reduction in pluripotency, and ultimately, the cessation of proliferation due to cellular senescence, mostly after passage 2–3 [7–10].

Herein, we describe a more effective and efficient culture methodology for ex vivo expansion of hAECs while retaining the innate nature of these epithelial cells. The success of the powerful epithelial cell culture technique, the Conditionally Reprogrammed Cells (CRC), has enabled the long-term culturing of primary mammalian epithelial cells (human, mouse, rat, dog, etc. tested to date) from various organs, including the prostate, breast, colon, and pancreas [11–14]. This method uses a Rho kinase inhibitor (Y-27632) ((+)-(R)-Trans-4-(1-aminoethyl)-*N*-(4-pyridyl) cyclo-hexanecarboxamide hydrochloride)) and J2 mouse fibroblast feeder cells to drive the indefinite proliferation of epithelial cells. These conditions, in particular the presence of Y-27632, allow the cells to bypass replicative senescence and become “conditionally” immortal without detectable cell crisis [11, 12, 14–16]. In our previous observations, these cultural conditions also protect cells against genomic instability, resulting in cell lines with morphological and genetic profiles similar to the initial primary cells [13].

## Materials and methods

### Placenta collection, amniotic membrane processing and hAECs isolation

Human placentae were donated by patients who underwent elective cesarean sections in the OB/GYN Department at Georgetown University Hospital Medical Center. All placental samples were de-identified and otherwise considered medical waste. No subject recruitment or consent was required for this study.

**Fig. 1:**
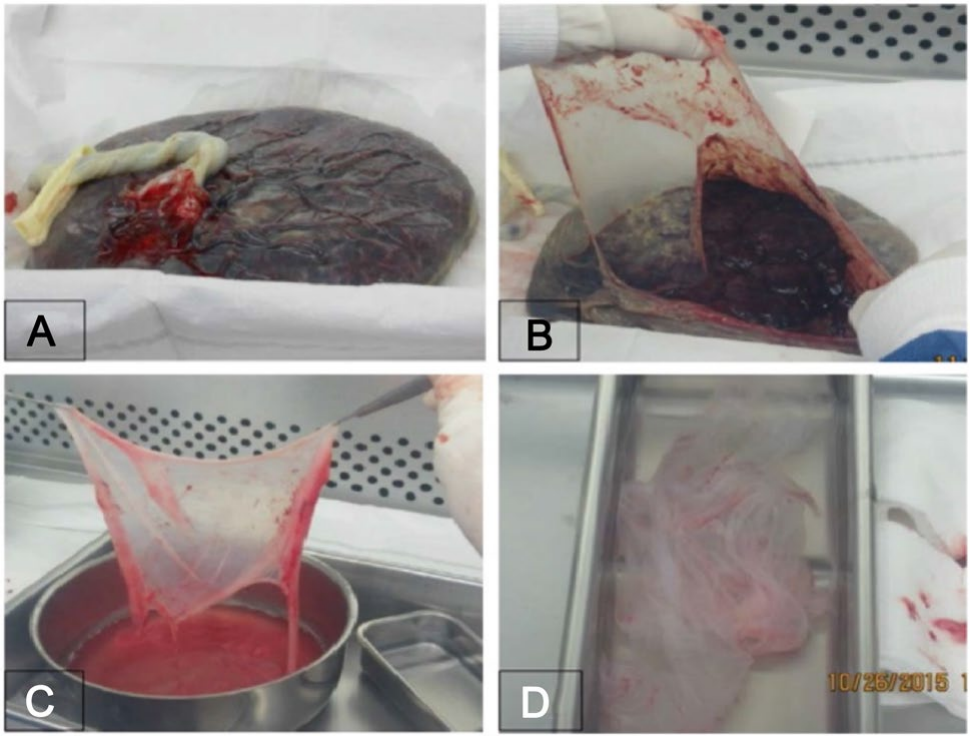
Processing amniotic membranes using sterile techniques. **A** Placenta examination, **B** amniotic membrane peeling, **C** blood removal, **D** fine trimming

**Fig. 2:**
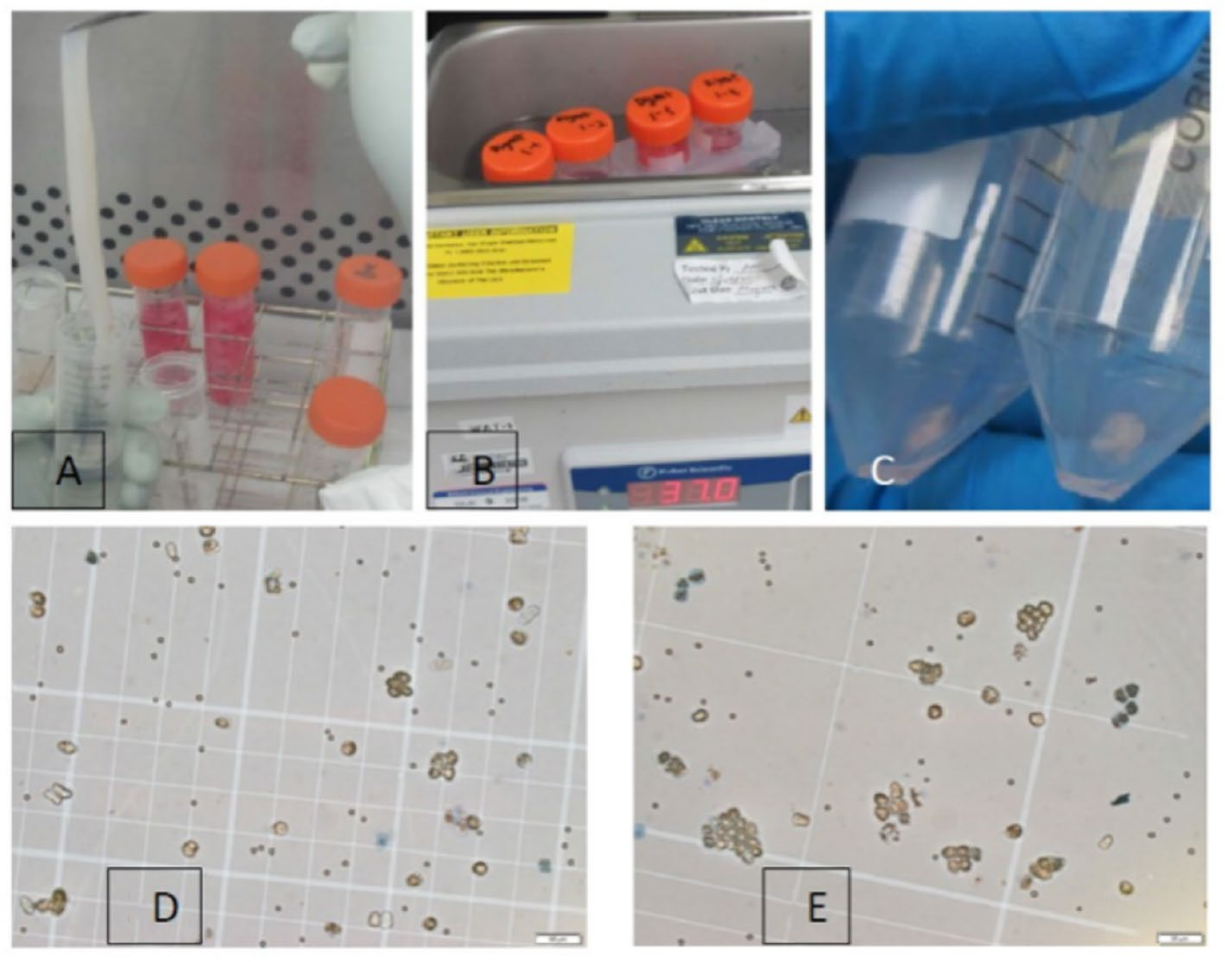
Human amniotic epithelial cell isolation. **A** Pre-digestion with HBSS, **B** Trypsin/EDTA digestion at 37 °C, **C** pellet by centrifugation, **D**, **E** cell counting

After collection, placental tissue was placed in containers with an ice-cold HBSS buffer and was transported immediately to the laboratory. For amniotic membrane isolation, the amniotic membrane was first dissected aseptically in a biological safety cabinet and then digested with 0.05% Trypsin/EDTA at 37 °C (Fig. 1A, B). The isolation of hAECs was performed following the protocol “Isolation of Amniotic Epithelial Stem Cells” [17]. The process of digestion was tightly monitored via microscopy as described in the protocol. In brief, the amnion layer was mechanically separated from the chorion layer (Fig. 1C) and washed several times with calcium and magnesium-free phosphate-buffered saline (PBS) to remove blood (Fig. 1D). To dissociate amniotic epithelial cells, the amniotic membrane (AM) was incubated at 37 °C with 0.05% trypsin containing 0.53 mM EDTA (Life Technologies, Grand Island, NY, USA). The digesta from the first 10 min of the trypsin digestion were discarded to exclude debris. The cells from the second and third 40-min digestions were pooled and washed three times with PBS (Fig. 2A–C). The parameters that were considered and calculated carefully in the isolation process are summarized in Table 1. Cell viability and quantity were determined after the isolation (Fig. 2D). The sterility of the cultures was determined by seeding 10 μl of cells in an LB plate and incubation at 37 °C.

**Table 1:** Isolation summary for hAECs isolated from the placenta

Placenta	Time calculations								
	Placenta waiting in OR (dry room temp)	Total time HBSS 4 °C	Peel and dip	Pre-digestion	1st digestion	2nd digestion	Isolation centrifuge (200 × g 10 min 4 °C)	Total process time	Total hAEC number (× 10^6^)
1	0:45	0:51	0:25	0:16	0:42	0:42	0:46	3:03	15:5
2	0:12	1:30	0:59	0:16	0:40	0:40	0:58	6:12	32:8
3	0:04	1:10	0:28	0:24	0:40	0:40	0:47	4:16	47:2

*OR* Operation room, *hAECs* human amniotic epithelial cells

### Expansion of hAECs using the CRC technology

The freshly isolated hAECs were cultured or cryopreserved immediately. Basic CRC conditions were used as defined previously [13–15], where CRC conditioned medium (CM) was used in the presence of 10% fetal bovine serum (FBS). The CM per se is F media that have been conditioned by the J2 cells for 3 days followed by filtration to remove debris and dead J2 cells and then mixed with fresh F media, 3:1 (v/v). The fresh *F* media are constituted of F-12 Nutrient Mixture (Ham)-DMEM (Invitrogen), 5% human serum (HS) (GIBCO) or 10% FBS (GIBCO), 0.4 μg/ml hydrocortisone (Sigma), 5 μg/ml insulin (Sigma), 8.4 ng/ml cholera toxin (Sigma), 10 ng/ml EGF (Invitrogen) and 24 μg/ml adenine (Sigma) with the addition of 5–10 μM Y-27632 (Enzo Life Sciences). Additional fresh 5uM Y-27632 was added to F media before use. All cells were maintained at 37 °C in a humidified incubator with 5% CO_2_. Uncultured cells and cells from every passage were cryopreserved using CRC cryopreservation media that is 90% HS or FBS, 10% DMSO and 5 mM Y-27632.

The cells were plated in CRC medium in collagen-coated flasks. Collagen-coated flasks for cell culture were prepared manually in the laboratory. The collagen solution (2:1 molecular grade water: collagen) was added into pre-sterilized flasks (300 μl for 25 cm^2^ flask; 500 μl for 75 cm^2^ flask) and spread evenly utilizing a sterilized scraper and quick hand rotations. The coated flasks were left in a sterile fume hood for 30 min and were then wrapped with parafilm sealing film and stored at 4 °C until use. Flasks were rinsed with PBS prior to use.

To culture cryopreserved hAECs, the samples were thawed, centrifuged at 1500 RPM for 5 min, and re-dissolved in either F media with FBS or F media with HS. hAECs were then plated in collagen-coated flasks in either F medium supplemented with 10% FBS or 5% HS. The hAECs growth was observed under the microscope daily and cells were passaged once they reach ~ 80% confluency or above. For passaging, media were aspirated, and cells were rinsed with PBS and treated with 0.05% trypsin or TrypEDTA solution in 37 °C incubator for 2 min. After two minutes, cultures were observed by phase microscopy and gently tapped to further detach cells. Detached cells were collected centrifuged at 1500 rpm for 5 min and re-suspended in either F medium supplemented with FBS or HS. Cells were then re-plated and passage numbers were recorded for each sample.

Population doubling experiments were performed from hAECs isolated from each sample to record and monitor their growth as reported previously [12, 18]. Briefly, cells were grown to confluency and were trypsinized, counted, and plated in a 1:4 ratio. The cell viability was determined using trypan blue dye exclusion staining. Cell counting was performed using a hemocytometer as previously described [14, 19]. Passage numbers and dates were logged for each sample to track population doubling times.

### Initial characterization of hAECs-CRC

Next, we next performed the initial characterization of freshly isolated uncultured hAECs, cultured hAECs-CRC and cryopreserved cells using microscopy, immunoblotting and flow cytometry. We analyzed the expression levels of pluripotent stem cell markers and immunogenic markers via immunoblotting and flow cytometry, respectively.

### Immunoblotting

Protein extracts from freshly isolated cultured and cryopreserved cells were separated on 4–20% tris–glycine gels and electro-blotted onto PVDF membranes as previously described [15, 19, 20]. Antibodies against REX-1 (Abcam, #ab175431), SOX-2 (Cell Signaling, #3579), OCT-4 (Cell Signaling, #2750), c-Myc (Abcam, #ab32072), NANOG (Cell Signaling, #4903), E-cadherin (Cell Signaling, #3195), cytokeratin 17/19 (Cell Signaling, # 3984), KLF-4 (Cell Signaling, 12,173) and β-actin (Cell Signaling, #4967) were purchased from indicated companies. Densitometry was performed using ImageJ analysis software (NIH, Bethesda, MD) as previously described [21].

### Flow cytometer analysis of stem cell markers

Immunophenotyping of hAECs was performed by flow cytometry according to our previously published protocols [22, 23]. After isolation hAECs were washed in cold PBS with 2% heat-inactivated FBS (stain buffer) and incubated for 30 min in staining buffer containing manufacturer-recommended concentrations of either FITC or PE-labeled antibodies. The isotype-matched antibodies were used as negative controls.

### Production of GFP-P2A-luc-containing lentiviral vector

The hAECs were infected with a puromycin-selectable lentivirus (pLV[Exp]-EGFP:T2A:Puro-EF1A > Luciferase) expressing luciferase and green fluorescent protein (GFP) designed using vector builder (VB160314-1002hdt) and stably selected with puromycin (Promega). The flow cytometry was performed in the Georgetown-Lombardi Flow Cytometry Shared Resource to verify and quantify receptor expression. The PerkinElmer In Vivo Imaging System (IVIS) in the Georgetown-Lombardi Preclinical Imaging Research Laboratory was used to quantify luciferase and GFP expression before implantation.

### Tolerability and stability of hAECs-CRC in vivo

To check the tolerability and stability of cells against the immune system, hAECs expressing GFP were injected subcutaneously into the right or left flanks of Athymic NCI/Nude mice (*n* = 2) and C57BL6 mice (*n* = 2). Approximately 3 × 10^5^ cells were injected into each flank. All animal procedures were conducted according to a protocol approved by the Georgetown IACUC (protocol # 2016–114). For the imaging experiments, mice were anesthetized with 1–3% Isoflurane. We then performed chemiluminescence imaging on a PerkinElmer IVIS Lumina III in the Georgetown-Lombardi Preclinical Imaging Research Laboratory by injecting the mice with D-luciferin (100ul, PerkinElmer 122,799 SBF1) to confirm the presence and viability of the tumor on days 2, 5 and 42.

## Results

We first established the morphology, purity and viability of freshly isolated hAECs via microscopy. The parameters involved in the isolation process are summarized in Table 1. In addition, the outcome and benchmark characteristics of the three step-isolation processes are also summarized in Table 1. Importantly, we were able to significantly enhance hAECs yields per placenta over time (from 15.5 million hAECs for placenta #1 to 47.2 million hAECs for placenta #3) highlighting the increased proficiency in the isolation of hAECs. The viability of hAECs from all isolations was greater than 90%. As noted in Table 1, the timely collection of placentae from the operation room (OR) (dry, room temperature) and immediate shifting to proper storage conditions significantly improved the yield of the cells. The total yield increased from 15.5 × 10^6^ (sample 1) to 47.2 × 10^6^ (sample 2) when the waiting time in OR was reduced from 00:45 h to 00:04 h, respectively. Of note, the total ischemic time before the start of the peel and dip process was higher for sample 2 (1:10 h) as compared to sample 1 (00:51 h).

**Fig. 3:**
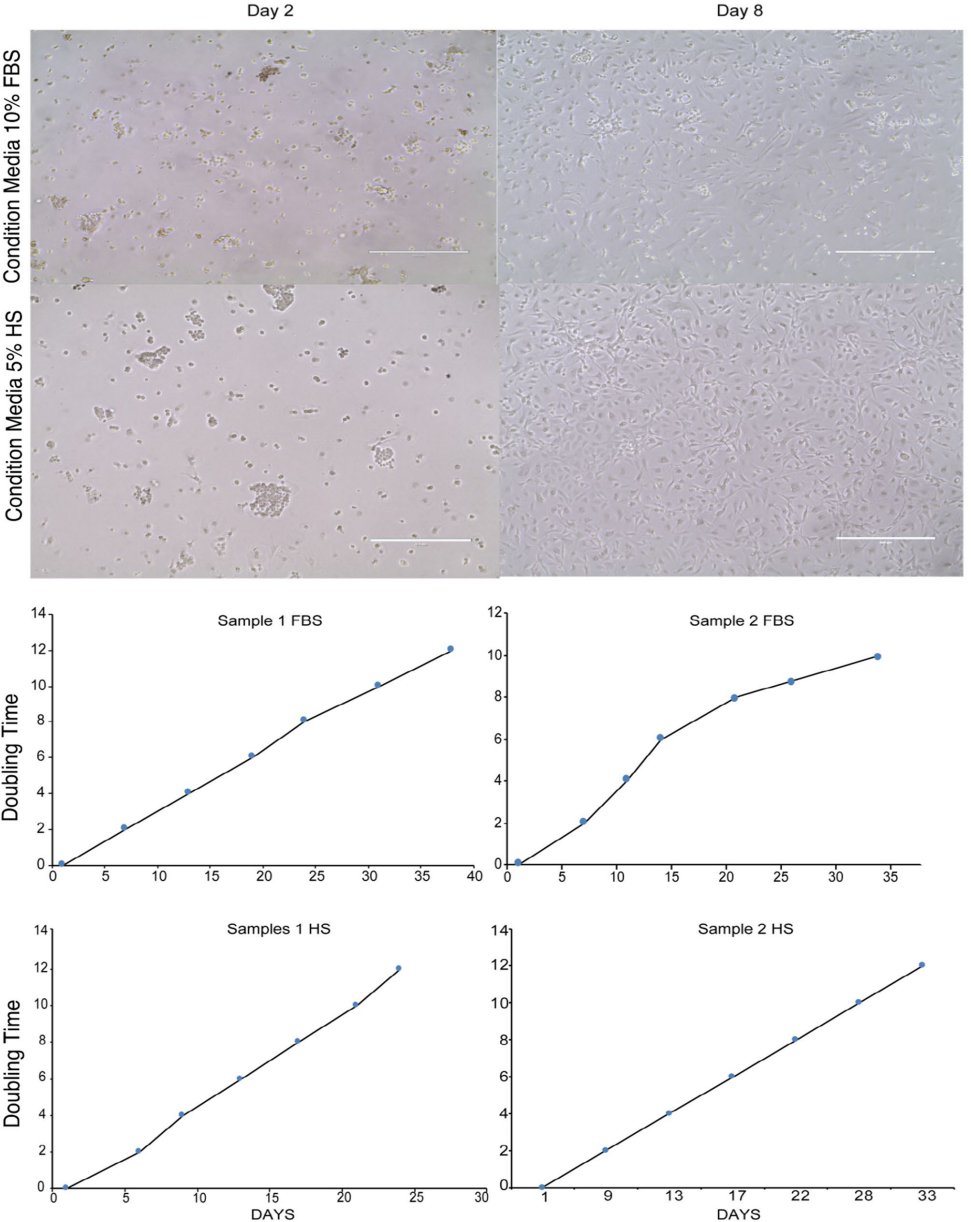
Human Amniotic epithelial cell expansion. **A** Human amniotic epithelial cells expanded under CRC conditions from days 2–8. Representative samples of hAEC-CRC in condition media supplemented with FBS and human serum (HS). **B**, **C** Population doubling experiments show the proliferative potential of two representative samples of hAECs-CRC in FBS (**B**) and HS (**C**)

The freshly isolated hAECs were cultured in the CRC culture conditions in the presence of 10% FBS or 5% HS and sustained their epithelial morphology (Fig. 3A). The hAECs were capable of vigorous proliferation in both FBS and xenogen-free (XF) HS conditions, thereby behaving in a manner consistent with all other primary epithelial cells that have been successfully expanded previously using CRC methodology (Fig. 3B, C). To evaluate the reversibility of conditional immortalization, we compared the growth of hAECs in CM with or without the Rho kinase inhibitor, Y27632 (Data not shown). As anticipated, a sharp decline in the growth of hAECs was observed in CM in the absence of Y-27632.

### Initial characterization of hAECs

Upon successful establishment of cryopreservation and CRC expansion protocols, we then defined and characterized their respective surface marker profile by immunochemistry and flow cytometry. The proliferation of the hAECs was assessed in CM supplemented with the human serum (HS) that is used for clinically approved stem cells.

**Fig. 4:**
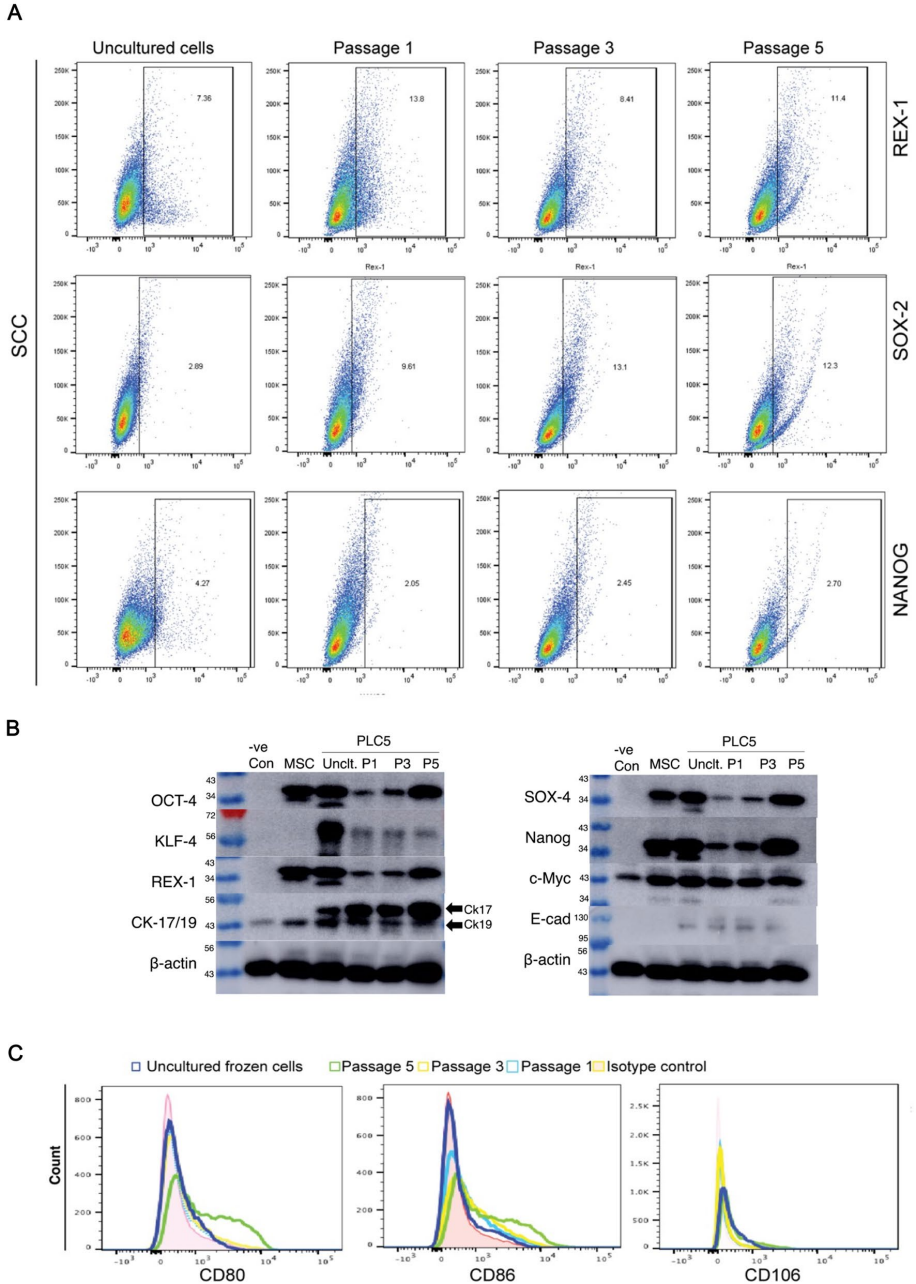
Human amniotic epithelial cell marker expression. **A**–**C** Flow cytometer and immunoblots for pluripotent and immunogenicity stem cell markers in uncultured hAECs and CRC expanded hAECs (hAECs-CRC) in HS. − *ve CON* negative control; *MSC* mesenchymal stem cells, *P* passage, *PLC5* placenta sample no. 5; *Unclt* uncultured cells

Next, we performed initial characterization of both uncultured hAECs and hAECs-CRC cultured via microscopy, immunoblotting and flow cytometry. We analyzed the expression levels of pluripotent stem cell markers including REX-1, SOX-2, OCT-4, KLF-4, c-Myc and NANOG via immunoblotting and flow cytometry (Fig. 4A, B). The human fibroblast cells were used in the negative control lane. The hAEC-CRCs were also assessed for the epithelial cell markers E-cadherin and cytokeratin 17/19. Mesenchymal stem cells (MSC) were used as a negative control for epithelial cell-specific markers E-cadherin. Importantly, the hAEC-CRCs were positive for both the stem and the epithelial cell markers (Fig. 4A).

Since hAECs have been shown to be non-immunogenic and fail to elicit proliferative responses in human peripheral blood mononuclear cells (PBMCs) in vitro [24], we also profiled the hAECs-CRC for expression levels of co-stimulatory markers such as CD80, CD86 and CD105 (Fig. 4C). The expression levels of these remained remarkably similar to the uncultured freshly isolated hAECs.

### Tolerability and stability of hAECs-CRC in vivo.

**Fig. 5:**
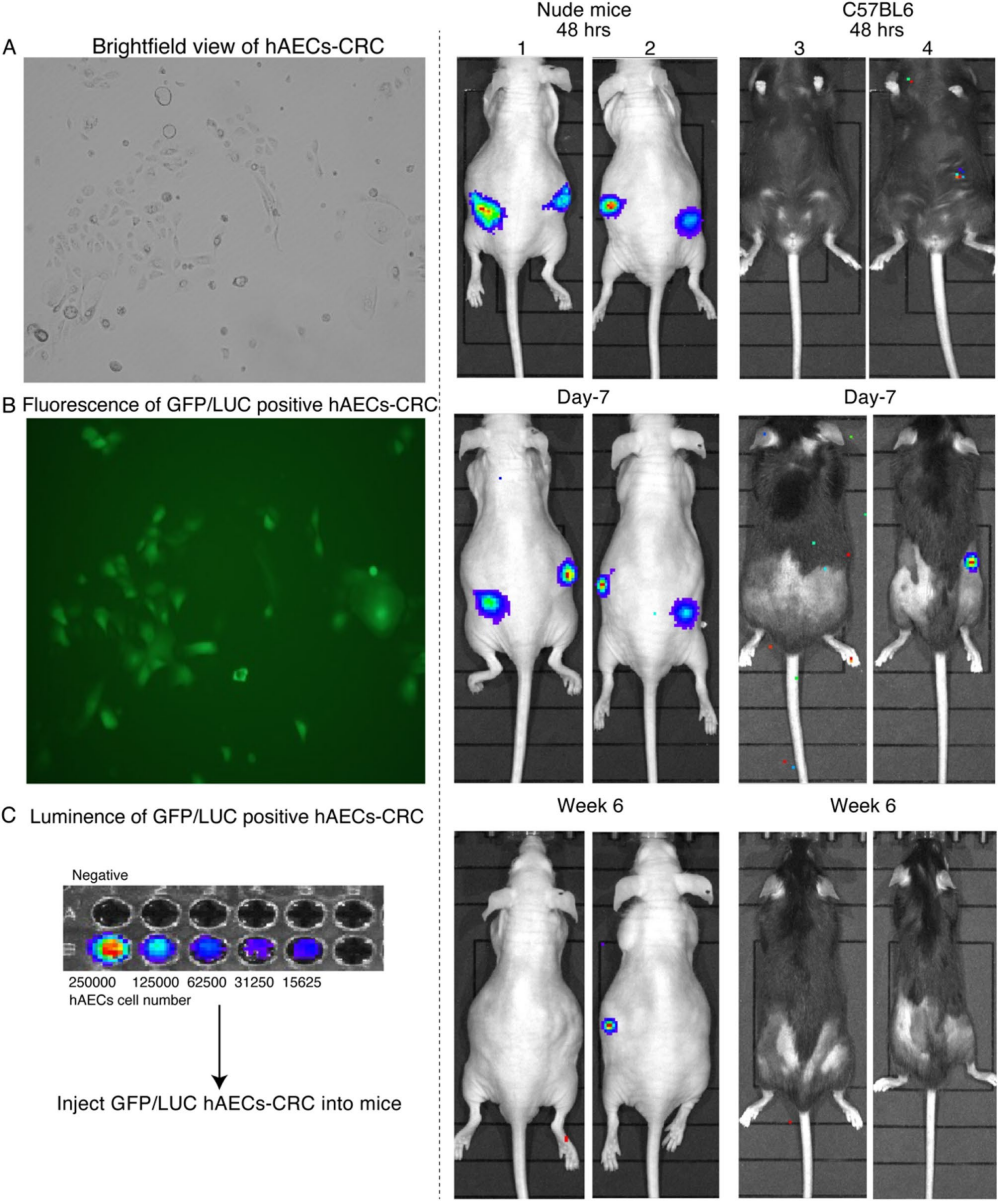
Selection of GFP/Luc lentivirus-labeled hAECs imaged by IVIS. **A**–**C** LUC and fluorescence images and quantification, **D** IVIS images of representative mice at 2, 5, and 42 days after transplantation of GFP/Luc positive hAECs-CRCs

hAECs-CRC were stably infected with GFP-luciferase (Fig. 5A–C). The hAECs-CRC were injected into flanks of immuno-suppressed Athymic Nude mice (which lack T cells) and immuno-competent mice and were supported seven days following initial injection, with continued fluorescent expression six weeks days following injection in immuno-compromised mice and 7 days in C57BL6 mice (Fig. 5D). The expression of hAECs was stronger in immuno-compromised the Athymic Nude mice, which retain an innate immune system and B cell development than in immuno-competent C57BL6 mice. There was no evidence of inflammation, immune rejection or tumor formation in either mouse model.

## Discussion

While the conditions that promote the indefinite proliferation of hAECs in XF conditions have not been adequately explored, there are several recent studies that address possible improvements in both cryopreservation and expansion of hAECs [6]. In this study, we demonstrated that hAECs are amenable to ex vivo expansion under CRC growth conditions in XF conditions.

As discussed in a recent study, several factors affect the isolation, yield and purity of hAECs [6]. All phases of sample collection are vulnerable to errors; therefore, precise and timely handling is critically important. Consistent with other studies, a substantial decrease in ischemic time and an increase in the number of cells suggests that quick transfer of the tissue to proper storage conditions for efficient subsequent isolation procedure is critical [25–27]. Using the CRC methodology, we were able to expand hAECs up to passage 8 in F medium supplemented with either FBS or HS, which is significant given the estimation that culture up to passage 5 is estimated to be necessary for therapeutic application for transplantation in one patient [28, 29]. The use of human serum (in DMEM/F12) has been reported previously [10]; however, the cells plateaued at passage 2, and no molecular characterization was performed on the cells cultured in human serum.

We have evaluated the impact of various cryopreservation media on stem cell markers and post-thaw viability in a recent comparative study [6]. As compared to commercially available and other tested cryopreservation media (e.g., amniotic fluid, CryoStor CS10, Stem Cell banker), cryopreservation media containing human serum used in this study (see Methods) also provides an easy and promising solution to preserve these cells effectively. Both the cryopreserved and CRC-cultured hAECs maintained important markers of pluripotency, OCT-4 and SOX-2 successfully up until passage 5, though the expression of stem markers appeared to decline with subsequent passaging.

Immunogenicity and tumorigenicity have been primary concerns for the clinical translation of stem cell products. To date, the failure of many stem cell therapy clinical trials occurred due to immune rejection or tumor generation or tumor promotion [30, 31]. Our in vivo analyses demonstrated that hAECs cultured under CRC conditions were capable of transplantation into both immune-compromised and immuno-competent murine models without rejection from the host immune system, as there was no detected immune response following 6 weeks in immune-compromised and seven days after injection in the C57BL6 mice. Our results show that the CRC-hAECs culture method retains the non-immunogenic nature of these cells, thus addressing the issue of immunogenicity after stem cell transplantation. hAECs proliferation, as hAECs are amenable to in vitro proliferation under CRC growth conditions, can be supported in an immuno-competent murine model following culture in CRC conditions.

Our data strongly suggest that the CRC culture conditions do not affect the pluripotent, tumorigenic or immunogenic properties of hAECs. Provided that hAECs can be cultured and expanded in vitro while also maintaining their multi-tissue-regenerative properties, the conditions we have developed may provide a milestone in cell transplantation allowing a single donor to produce a sufficient number of cells for clinical use. Future studies addressing the genomic stability and exploring the differentiation potential of hAECs in humanized CRC conditions are underway and would further strengthen the CRC approach and its applicability.

## Conclusion

The application of the CRC method for the rapid expansion of hAECs is a key step towards the future use of hAECs for therapeutic application as stem-like cells for the replacement of tissues damaged by disease or injury as well as aging. We speculate that our CRC-based cell culturing methodology will provide an easy and efficient means to expand and cryopreserve hAECs-CRC without compromising their privileged epithelial characteristics*.*

## Supplementary Information

Below is the link to the electronic supplementary material.

Supplementary file1 (DOCX 920 kb)

## Data Availability

The data sets used and analyzed during the current study are available from the corresponding author upon reasonable request.

## Abbreviations

AM: Amniotic membrane
CRC: Condition cell reprogramming technology
CM: Conditioned media
DMEM: Dulbecco’s modified eagle medium
ESCM: Epithelial cell surface markers
FBS: Fetal bovine serum
GFP: Green fluorescent protein
hAECs: Human amniotic epithelial cells
HLA: Human leukocyte antigen
HS: Human serum
OR: Operation room
P: Passage
XF: Xenogeneic free
