# Supplementary material for: Expansion of human amniotic epithelial cells using condition cell reprogramming technology
Source: Hum Cell. 2022 Dec 31;36(2):602–11. doi: 10.1007/s13577-022-00849-4 (PMC9947022; doi:10.1007/s13577-022-00849-4)
Supplement: Supplementary file 1 — Supplementary file1 (DOCX 920 kb) [file 13577_2022_849_MOESM1_ESM.docx]

## **Expansion of human amniotic epithelial cells using** **condition cell reprogramming technology**

Aisha Naeem^1,2#*^, Muhammad Umer Choudhry^1#^, Alex Kroemer^3^, Simone Wahnschafft^1^, Wanxing Cui^4^, Chris Albanese^1,5,6^

^#^These authors contributed equally.

^1^Department of Oncology, Lombardi Comprehensive Cancer Center, Georgetown University Medical Center, Washington, DC.

^2^Health Research Governance Department, Ministry of Public Health, Qatar.

^3^MedStar Georgetown Transplant Institute, MedStar Georgetown University Hospital, Center for Translational Transplant Medicine, Georgetown University, Washington, DC 20007, USA

^4^MedStar Georgetown University Hospital, Washington, DC

^5^Department of Radiology, Georgetown University Medical Center, Washington, DC.

^6^Center for Translational Imaging, Georgetown University Medical Center, Washington, DC.

*Corresponding author: Aisha Naeem; anaeem@moph.gov.qa/an594@georgetown.edu

Address: Human Research Governance Department, Ministry of Public Health, Doha, Qatar/Department of Oncology, Lombardi Comprehensive Cancer Center, Georgetown University Medical Center, Washington, DC.

Figure 1: Uncropped western blot images.
